# Serum Uric Acid Might Be Positively Associated With Hypertension in Chinese Adults: An Analysis of the China Health and Nutrition Survey

**DOI:** 10.3389/fmed.2021.755509

**Published:** 2022-01-05

**Authors:** Yingdong Han, Kaidi Han, Xinxin Han, Yue Yin, Hong Di, Juan Wu, Yun Zhang, Xuejun Zeng

**Affiliations:** ^1^Division of General Internal Medicine, Department of Primary Care and Family Medicine, Peking Union Medical College Hospital, Chinese Academy of Medical Sciences, Beijing, China; ^2^Department of Critical Care Medicine, The Affiliated Yantai Yuhuangding Hospital of Qingdao University, Yantai, China

**Keywords:** uric acid, hypertension, blood pressure, hyperuricemia, China health and nutrition survey

## Abstract

**Background:** Previous studies have clarified the relationship between serum uric acid (SUA) and hypertension; most of previous studies suggest that elevated uric acid levels are associated with an increased risk of hypertension, while in China, there are relatively few studies to explore above association. The objective of this longitudinal study is to investigate the correlation of SUA and hypertension in Chinese adults with a nationwide large-scale sample.

**Methods:** Data from the China Health and Nutrition Survey 2009, 2011, and 2016 were used; a total of 8,469 participants (3,973 men and 4,496 women) were involved. This study was conducted separately by gender. Clinical characteristics of the participants among different uric acid groups are compared. The binary logistic regression analysis was conducted to examine the association between SUA and hypertension. Restricted cubic spline analysis with three knots of the SUA concentration were used to characterize the dose-response relationship. Additionally, we compared the incidence of hypertension in the different baseline uric acid groups during follow-up in 2011 and 2015.

**Results:** After the covariates were fully adjusted, we found that elevated uric acid levels were correlated with increased risk of hypertension in both males (*p* < 0.01) and females (*p* < 0.01). With 2-year or 6-year of follow-up, we found participants with higher baseline uric acid levels had a higher incidence of hypertension (*p* < 0.01). In stratified analysis by obesity, above relationship remained significant in nonobesity population (males: *p* < 0.05, females: *p* < 0.01) and became nonsignificant in obesity people. In stratified analysis by age, above positively correlation remained significant in middle-aged men (*p* < 0.05) and elderly women (*p* < 0.01). Restricted cubic spline revealed the dose-response relationship between SUA and hypertension; we also found that above relationship was much stronger in females.

**Conclusion:** This study suggests that elevated SUA levels might be positively associated with an increased risk of hypertension in general Chinese adults.

## Introduction

Uric acid is the end product of purine nucleotide metabolism. Fatty meat, organ meat, seafood, and fructose are main exogenous sources, which can increase serum uric acid (SUA). Elimination of uric acid occurs mainly via kidney and intestine. Diminished excretion and overproduction of uric acid are the basis of hyperuricemia (1). A nationally representative survey of the United States showed that the mean SUA was 6.04 mg/dL among men and 4.79 mg/dL among women and prevalence of hyperuricemia was 20.2 and 20.0%, respectively (2). In China, a meta-analysis included 44 studies from 2000 to 2014 and found that the prevalence of hyperuricemia was 13.3% (3). The prevalence of both gout and hyperuricemia has increased over the past two decades (4). Elevated uric acid levels are definitely associated with an increased risk of gout, as the deposition of urate crystals in joints can cause acute inflammatory response, in addition to joint involvement; elevated uric acids are linked with renal disease and metabolic disorders including hypertension, type 2 diabetes, obesity, and metabolic syndrome (5–8). Previous studies have shown that elevated SUA is an independent risk factor for cardio-cerebrovascular (9) and all-cause mortality. Compared with untreated patients with hyperuricemia, those who receive urate-lowering therapy have a lower risk of all-cause mortality (10).

Hypertension, one of the most significant preventable risk factors for cardio-cerebrovascular disease, renal disease, and cognitive dysfunction, affects millions of people and is a leading cause of disability and all-cause mortality worldwide (9, 11). The prevalence of elevated blood pressure (BP) is rising owing to aging and exposing to unhealthy lifestyle such as lack of physical activity, excessive alcohol and sodium consumption, obesity, and low potassium intake (12, 13). In 2015, the estimated systolic BP (SBP) was 127.0 mm Hg in men and 122.3 mm Hg in women (12). Previous study showed that the global prevalence of hypertension in adults age 20 years and over increased from 25.9 (24.6–27.1%) in 2000 to 31.1% (30.0–32.2%) in 2010, which was higher in men (31.9%) and low-income and middle-income countries (14). Appropriate BP targets for different patients are currently topics of vigorous debate. Most guidelines recommended that BP should be reduced <140/90 mm Hg for most patients and <150/90 mm Hg for elderly patients. Successful prevention and treatment of hypertension, such as lifestyle changes and antihypertensive drugs, can reduce the disease burden, risk of cardiovascular events and all-cause mortality, and promote longevity in worldwide (13).

In 1870's, Mahomed et al. first hypothesized the association between SUA and hypertension; since then, numerous epidemiological studies have proved above hypothesis. Hyperuricemia is common among people with hypertension and prehypertension (15, 16). A meta-analysis found that the allopurinol therapy group had greater reduction in both SBP and diastolic blood pressure compared with the control group and in subgroup analysis, whether antihypertensive drugs were being administered, allopurinol significantly reduced SBP (17). However, there are relatively few studies on the relationship between uric acid and hypertension in Chinese population. Therefore, we performed this study in a nationally large-scale cohort of Chinese adults using data from the China Health and Nutrition Survey (CHNS) 2009 to explore the correlation between uric acid and hypertension and hoped it may provide some information for the prevention and treatment of hypertension.

## Materials and Methods

### Data Collection and Sample

**Figure 1:**
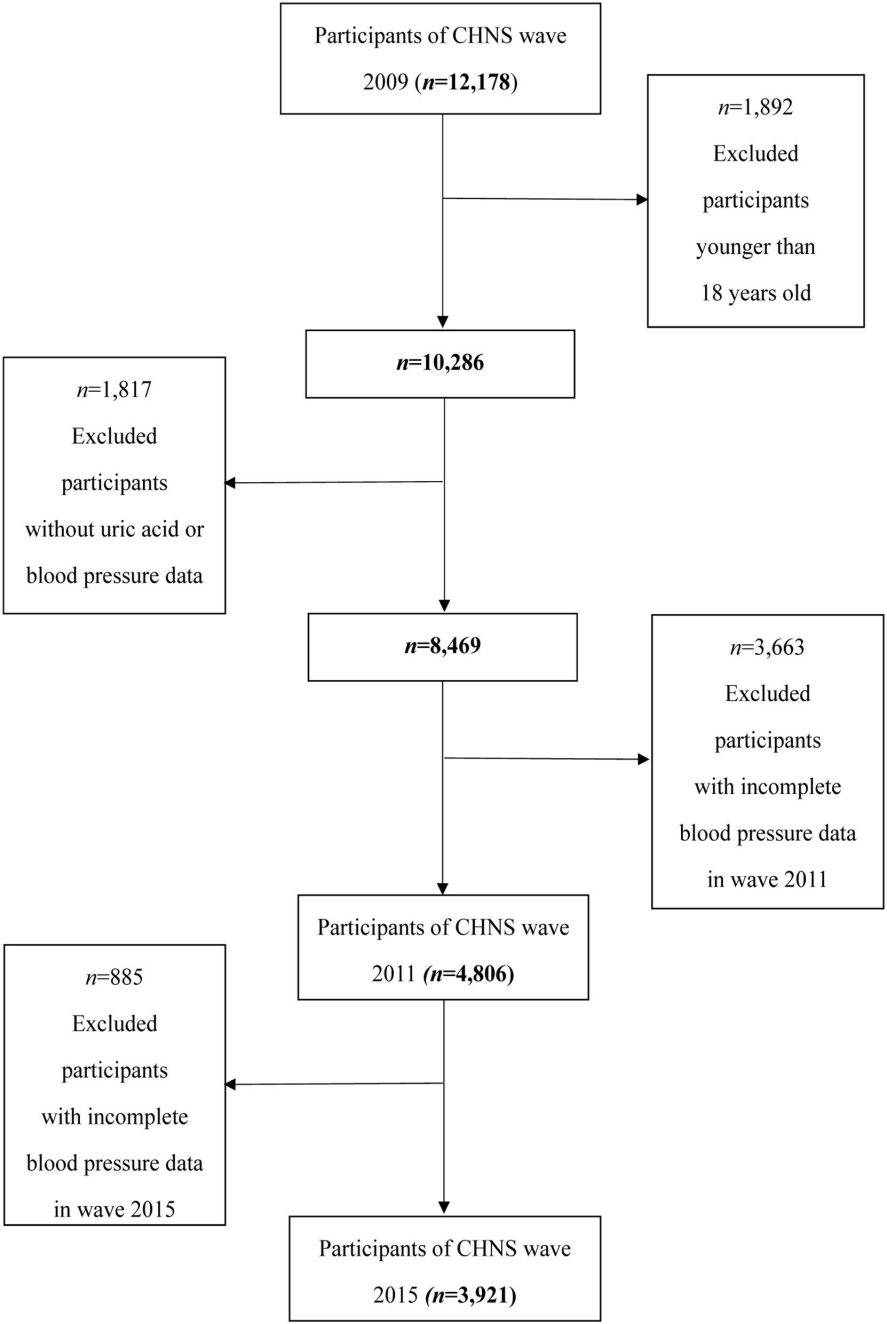
Flowchart of the screening process for the selection of eligible participants.

The CHNS, an international collaborative project between the Carolina Population Center at the University of North Carolina at Chapel Hill and the National Institute of Nutrition and Health at the Chinese Center for Disease Control and Prevention, is conducted by an international team of researchers whose backgrounds include nutrition, public health, economics, sociology, and demography (18). This study was conducted in 15 provinces and municipal cities, which is a large-scale sample of Chinese and can reflect the nutritional and health status of the Chinese people (19). In this study, we used the results of the 2009 survey because SUA collection occurs only in 2009 after excluding those who under 18 years old (*n* = 1,892) and those with incomplete uric acid data (*n* = 1,618) and hypertension information (*n* = 199). A total of 8,469 individuals (3,973 men and 4,496 women) were included in this study; of these participants, 4,806 participants with normal BP in 2009 were measured again in the 2011 follow-up and 3,921 participants were involved in the 2015 follow-up. The flowchart of the screening process is shown in Figure 1. A written informed consent was obtained from all the participants before they participated in this study. This study was in accordance with the ethical standards of the responsible committee on human experimentation (institutional or regional) and with the Helsinki Declaration of 1975.

### Study Variables

Serum uric acid is measured by Hitachi 7600 automated analyzer using an enzymatic colorimetric method. Triplicate measurements of BP were taken after 10 min at sitting and with at least 1 min between recordings with the use of mercury sphygmomanometer and means of the three BP measurements were used in the final analysis (20). Participants who met at least one of the following criteria: SBP ≥ 140 mm Hg or diastolic blood pressure ≥ 90 mm Hg, using antihypertensive drugs during the previous 2 weeks, or ever diagnosed with hypertension by a physician (21).

Potential confounding factors in this study include age; gender; race (Han and other race); body mass index (BMI) (underweight: <18.5 kg/m^2^, normal: between 18.5 and <24 kg/m^2^, overweight: between 24.0 and <28.0 kg/m^2^, or obesity ≥28 kg/m^2^) (22); residence location (urban and rural); education [lower than middle school (<6 years), middle school (6.1–9.0 years), high school (9.1–12 years), and college/university (>12 years)]; total energy intake (kcal/day); serum creatinine; triglyceride; total cholesterol; high-sensitivity C-reactive protein (hsCRP); fasting blood glucose; smoking status (never or ever); and drinking [yes (ever drank beer/alcohol the year before the examination) or no (did not drink beer/alcohol the year before the examination)]. History of diabetes is defined as self-reported diagnosed with diabetes.

### Statistical Analysis

All the statistical analyses were conducted with the SPSS software version 23.0 (IBM Corporation, Chicago, USA) and the R software version 4.1.0. (Core Team, Vienna, Austria). The level of SUA is quite different between men and women this study was conducted separately by gender. The normality of continuous variables was tested with the Kolmogorov–Smirnov normality test. Normally distributed variables were described with mean ± SD and non-normally distributed continuous variables were described with median (interquartile range). The median values among the different SUA groups were compared with the Kruskal–Wallis test. The chi-squared test was adopted to compare the percentages of categorical variables among the different SUA groups. The Bonferroni test was used for the intergroup comparison. SUA level in our analyses was modeled in quartiles: Q1 (SUA ≤ 4.86 mg/dl), Q2 (4.86 < SUA ≤ 5.73 mg/dL), Q3 (5.73 < SUA ≤ 6.79 mg/dL), and Q4 (SUA > 6.79 mg/dL) in males and Q1 (SUA ≤ 3.55 mg/dL), Q2 (3.55 < SUA ≤ 4.29 mg/dl), Q3 (4.29 < SUA ≤ 5.23 mg/dL), and Q4 (SUA > 5.23 mg/dL) in females, with the first quartile (Q1) as the reference group. Hyperuricemia is defined as SUA level ≥7 and ≥6 mg/dL in males and females, respectively (23). The binary logistic regression analysis was conducted to examine the association between SUA and hypertension. Age and race were adjusted in model 1 and model 2 was additionally adjusted for living location, BMI, alcohol consumption, smoking, diabetes, education, serum creatinine, glucose, triglyceride, total cholesterol, hsCRP, and total energy intake. Restricted cubic spline analysis with three knots of the SUA concentration was used to characterize the dose-response relationship in the logistic regression model 2. Additionally, we compared the incidence of hypertension in the different baseline uric acid groups during follow-up in 2011 and 2015. In addition, stratified analysis by age and obesity was performed to examine above association. A two-sided *p* < 0.05 was considered as statistically significant.

## Results

A total of 8,469 individuals (3,973 men and 4,496 women) were included in this study, with a mean ± SD age of 50.33 ± 15.08 years, with a mean ± SD SUA of 5.19 ± 1.78 mg/dL. 20.2% of male participants and 11.1% of female participants met the diagnostic criteria of hyperuricemia. 30.0% of men and 26.7% of women met the diagnostic criteria of hypertension. The clinical characteristics of participants among different SUA levels are shown in Tables 1, 2. We found a greater proportion of hypertension and obesity in both male and female participants belonged to the highest quartile of SUA. With the increasing of uric acid quartiles, the median of triglyceride and creatinine increased gradually in male participants. With the increasing of uric acid quartiles, the median of serum glucose, triglyceride, total cholesterol, and creatinine increased gradually in female participants.

**Table 1:** Clinical characteristics of the study population disaggregated by quartiles of serum uric acid according to the China Health and Nutrition Survey (CHNS) 2009 (males = 3,973).

	**Uric acid quartiles**				
	**Group 1**	**Group 2**	**Group 3**	**Group 4**	***P* Value**
Number of subjects	1000	1007	984	982	
Age (year)^‡^	53 (20)	50 (21)	50 (24)	50 (21)	0.013
Race (%)^†^					<0.01
Han	911 (91.3)	887 (88.3)	872 (88.8)	859 (88.0)	
Others	87 (8.7)	117 (11.7)	110 (11.2)	117 (12.0)	
Body mass index (%)^†^					<0.01
Underweight: <18.5(kg/m^2^)	91 (9.3)	60 (6.1)	59 (6.1)	28 (2.9)	
Normal: BMI≥18.5 and <24(kg/m^2^)	594 (60.4)	598 (60.7)	476 (49.5)	403 (41.9)	
Overweight: BMI≥24 and <28(kg/m^2^)	254 (25.8)	260 (26.4)	331 (34.4)	392 (40.8)	
Obesity: BMI≥28(kg/m^2^)	44 (4.5)	67 (6.8)	95 (9.9)	138 (14.4)	
Cholesterol (mmol/L)^‡^	4.57 (1.13)	4.66 (1.15)	4.73 (1.25)	5.03 (1.32)	<0.01
Triglyceride (mmol/L)^‡^	0.98 (0.70)	1.16 (0.87)	1.38 (1.19)	2.16 (2.43)	<0.01
Glucose (mmol/L)^‡^	5.05 (0.96)	5.07 (0.89)	5.14 (0.92)	5.35 (1.14)	<0.01
Creatinine (μmol/L)^‡^	88 (16)	94 (15)	96 (17)	100 (18)	<0.01
Hs-CRP (mg/L)^‡^	1.0 (2.0)	2.0 (2.0)	2.0 (1.0)	2.0 (2.0)	<0.01
Living location (%)^†^					<0.01
Urban	305 (30.5)	294 (29.2)	326 (33.1)	377 (38.4)	
Rural	695 (69.5)	713 (70.8)	658 (66.9)	605 (61.6)	
Serum uric acid (mg/dL)^‡^	4.27 (0.70)	5.29 (0.45)	6.22 (0.52)	7.73 (1.33)	<0.01
Total energy intake (kcal/d)^‡^	2260 (950)	2276 (858)	2294 (781)	2279 (802)	0.809
Education (%)^†^					<0.01
Lower than middle school	385 (38.6)	345 (34.3)	322 (32.8)	290 (29.6)	
Middle school	390 (39.1)	405 (40.3)	364 (37.0)	351 (35.9)	
High school	135 (13.5)	138 (13.7)	132 (13.4)	143 (14.6)	
College and above	87 (8.7)	117 (11.6)	165 (16.8)	195 (19.9)	
Hypertension (%)^†^	253 (25.3)	280 (27.8)	300 (30.5)	360 (36.7)	<0.01
Systolic blood pressure (mmHg)^‡^	121.3 (16.7)	121.3 (22.84)	122.7 (22.0)	123.3 (24.0)	<0.01
Diastolic blood pressure (mmHg)^‡^	80.0 (11.3)	80.0 (15.1)	80.7 (14.1)	80.7 (16.7)	<0.01
Diabetes (%)^†^	36 (3.6)	26 (2.6)	23 (2.3)	42 (4.3)	0.050
Drinking status (%)^†^					0.028
Yes	587 (58.7)	585 (58.1)	585 (59.5)	629 (64.1)	
No	413 (41.3)	422 (41.9)	399 (40.5)	353 (35.9)	
Ever smoking (%)^†^	611 (61.1)	647 (64.3)	593 (60.3)	590 (60.1)	0.191

*Data are number of subjects (percentage) or medians (inter quartile ranges)*.

† *Chi-square test was used to compare the percentage among participants in different groups*.

‡ *Kruskal-Wallis test was used to compare the median values among participants in different groups*.

*Uric acid quartiles: Group 1 (SUA ≤ 4.86 mg/dL), Group 2 (4.86 < SUA ≤ 5.73 mg/dL), Group 3 (5.73 < SUA ≤ 6.79 mg/dL), Group 4 (SUA > 6.79 mg/dL)*.

**Table 2:** Clinical characteristics of the study population disaggregated by quartiles of serum uric acid (SUA) according to the CHNS 2009 (females = 4,496).

	**Uric acid quartiles**				
	**Group 1**	**Group 2**	**Group 3**	**Group 4**	***P* Value**
Number of subjects	1130	1126	1131	1109	
Age (year)^‡^	45 (18)	48 (19)	52 (21)	57 (20)	<0.01
Race (%)^†^					0.972
Han	997 (88.7)	994 (88.4)	1000 (88.8)	977 (88.3)	
Others	127 (11.3)	131 (11.6)	126 (11.2)	130 (11.7)	
Body mass index (%)^†^					<0.01
Underweight: <18.5(kg/m^2^)	83 (7.4)	88 (8.0)	65 (5.8)	47 (4.3)	
Normal: BMI≥18.5 and <24(kg/m^2^)	708 (63.3)	631 (57.1)	574 (51.5)	472 (43.2)	
Overweight: BMI≥24 and <28(kg/m^2^)	264 (23.6)	306 (27.7)	359 (32.2)	362 (33.1)	
Obesity: BMI≥28(kg/m^2^)	63 (5.6)	81 (7.3)	117 (10.5)	212 (19.4)	
Cholesterol(mmol/L)^‡^	4.52 (1.24)	4.64 (1.27)	4.95 (1.33)	5.16 (1.42)	<0.01
Triglyceride(mmol/L)^‡^	0.95 (0.62)	1.11 (0.76)	1.33 (1.05)	1.80 (1.63)	<0.01
Glucose(mmol/L)^‡^	4.92 (0.73)	5.00 (0.79)	5.13 (0.86)	5.37 (1.04)	<0.01
Creatinine (μmol/L)^‡^	73 (11)	76 (11)	79 (13)	84 (17)	<0.01
Hs-CRP (mg/L)^‡^	1.0 (1.0)	1.0 (2.0)	1.0 (2.0)	2.0 (3.0)	<0.01
Living location (%)^†^					<0.01
Urban	337 (19.8)	374 (33.2)	364 (32.2)	417 (37.6)	
Rural	793 (70.2)	752 (66.8)	767 (67.8)	692 (62.4)	
Serum uric acid (mg/dL)^‡^	3.11 (0.54)	3.93 (0.35)	4.71 (0.47)	5.94 (1.11)	<0.01
Total energy intake (kcal/d)^‡^	1905 (735)	1893 (736)	1923 (715)	1904 (683)	0.307
Education (%)^†^					<0.01
Lower than middle school	532 (47.2)	538 (47.8)	590 (52.2)	656 (59.2)	
Middle school	376 (33.3)	336 (29.9)	308 (27.3)	266 (24.0)	
High school	112 (9.9)	131 (11.6)	110 (9.7)	86 (7.8)	
College and above	108 (9.6)	120 (10.7)	122 (10.8)	100 (9.0)	
Hypertension (%)^†^	190 (16.8)	242 (21.5)	299 (26.4)	472 (42.6)	<0.01
Systolic blood pressure (mmHg)^‡^	118.7 (18)	119.3 (20.7)	120.0 (26)	129.3 (26)	<0.01
Diastolic blood pressure (mmHg)^‡^	77.3 (11.3)	78.7 (14)	80 (14.7)	80 (16)	<0.01
Diabetes (%)^†^	18 (1.6)	21 (1.9)	22 (1.9)	56 (5.0)	<0.01
Drinking status (%)^†^					0.379
Yes	98 (8.7)	90 (8.0)	113 (10.0)	94 (8.5)	
No	1032 (91.3)	1036 (92.0)	1018 (90.0)	1015 (91.5)	
Ever smoking (%)^†^	46 (4.1)	40 (3.6)	51 (4.5)	46 (4.1)	0.718

*Data are number of subjects (percentage) or medians (interquartile ranges)*.

† *The chi-squared test was used to compare the percentage among participants in the different groups*.

‡ *The Kruskal–Wallis test was used to compare the median values among participants in the different groups*.

*Uric acid quartiles: Group 1 (SUA ≤ 3.55 mg/dL), Group 2 (3.55 < SUA ≤ 4.29 mg/dL), Group 3 (4.29 < SUA ≤ 5.23 mg/dL), and Group 4 (SUA > 5.23 mg/dL)*.

The results of the binary logistic regression analysis between SUA levels and hypertension were shown in Tables 3, 4. In males, the crude odds ratios (ORs) with 95% CIs of hypertension were 1.14 (0.93–1.39), 1.29 (1.06–1.57), and 1.71 (1.41–2.07) in Q2, Q3, and Q4 vs. Q1 group of SUA, respectively. In model 1, after adjustment for age and race, the adjusted ORs with 95% CIs were 1.26 (1.02–1.55), 1.47 (1.19–1.82), and 2.00 (1.63–2.46) in Q2, Q3, and Q4 vs. Q1 group, respectively. In model 2, the multivariate-adjusted ORs with 95% CIs of hypertension were 1.19 (0.95–1.48), 1.25 (0.99–1.56), and 1.46 (1.15–1.85) in Q2, Q3, and Q4 vs. Q1 group, respectively. In females, the crude ORs with 95% CIs of hypertension were 1.34 (1.09–1.65), 1.76 (1.43–2.16), and 3.61 (2.97–4.39) in Q2, Q3, and Q4 vs. Q1 group, respectively. In model 1, after adjustment for age and race, the adjusted ORs with 95% CIs were 1.17 (0.93–1.47), 1.38 (1.10–1.72), and 2.24 (1.81–2.77) in Q2, Q3, and Q4 vs. Q1 group, respectively. In model 2, the multivariate-adjusted ORs with 95% CIs of hypertension were 1.07 (0.85–1.36), 1.13 (0.89–1.44), and 1.48 (1.16–1.89) in Q2, Q3, and Q4 vs. Q1 group, respectively. We observed a dose-response relationship between SUA levels and risk of hyperuricemia in both males and females (*p* < 0.05). In Table 4, participants in the hyperuricemia group had a higher risk of hypertension compared with participants in the normal group. In model 2, the multivariate-adjusted ORs with 95% CIs of hypertension were 1.30 (1.06–1.59) and 1.40 (1.11–1.78) in males and females, respectively.

**Table 3:** Weighted odds ratios (95% CIs) for hypertension of participants across quartiles of serum uric acid (SUA) (n = 8,469, males = 3,973, and females = 4,496).

	**Case/Participants**	**Crude^‡^**	**Model 1^‡^**	**Model 2^‡^**
Uric acid quartiles^†^				
Males				
Q1	1000/3973	1.00 (Ref.)	1.00 (Ref.)	1.00 (Ref.)
Q2	1007/3973	1.14 (0.93–1.39)	1.26 (1.02–1.55)^*^	1.19 (0.95–1.48)
Q3	984/3973	1.29 (1.06–1.57)^*^	1.47 (1.19–1.82)^**^	1.25 (0.99–1.56)
Q4	982/3973	1.71 (1.41–2.07)^**^	2.00 (1.63–2.46)^**^	1.46 (1.15–1.85)^**^
*P* for trend		*P* < 0.05	*P* < 0.05	*P* < 0.05
Uric acid quartiles^†^				
Females				
	1130/4496	1.00 (Ref.)	1.00 (Ref.)	1.00 (Ref.)
	1126/4496	1.34 (1.09–1.65)^**^	1.17 (0.93–1.47)	1.07 (0.85–1.36)
	1131/4496	1.76 (1.43–2.16)^**^	1.38 (1.10–1.72)^**^	1.13 (0.89–1.44)
	1109/4496	3.61 (2.97–4.39)^**^	2.24 (1.81–2.77)^**^	1.48 (1.16–1.89)^**^
*P* for trend		*P* < 0.05	*P* < 0.05	*P* < 0.05

† *Males: Q1 (SUA ≤ 4.86 mg/dL), Q2 (4.86 < SUA ≤ 5.73 mg/dL), Q3 (5.73 < SUA ≤ 6.79 mg/dL), and Q4 (SUA > 6.79 mg/dL)*.

*Females: Q1 (SUA ≤ 3.55 mg/dL), Q2 (3.55 < SUA ≤ 4.29 mg/dL), Q3 (4.29 < SUA ≤ 5.23 mg/dL), Q4 (SUA > 5.23 mg/dL)*.

‡ *Calculated using the binary logistic regression*.

*Model 1 adjusted for age and race*.

*Model 2 adjusted for age, race, living location, body mass index (BMI), alcohol consumption, smoking, diabetes, education, serum creatinine, glucose, triglyceride, total cholesterol, high-sensitivity C-reactive protein (hsCRP), and total energy intake*.

* *p < 0.05*;

** *p < 0.01*.

**Table 4:** The ORs (95% CIs) of hypertension for participants with hyperuricemia compared with normal uric acid level (*N* = 8,469, males = 3973, females = 4496).

	**Crude^‡^**	**Model 1^‡^**	**Model 2^‡^**
**Males**			
Normal uric acid	1.00 (Ref.)	1.00 (Ref.)	1.00 (Ref.)
Hyperuricemia	1.57 (1.34-1.85)^**^	1.70 (1.43-2.02)^**^	1.30 (1.06-1.59)^*^
**Females**			
Normal uric acid	1.00 (Ref.)	1.00 (Ref.)	1.00 (Ref.)
Hyperuricemia	3.02 (2.49-3.65)^**^	2.03 (1.65-2.50)^**^	1.40 (1.11-1.78)^**^

‡ *Calculated using binary logistic regression*.

*Model 1 adjusted for age and race*.

*Model 2 adjusted for age, race, living location, BMI, alcohol consumption, smoking, diabetes, education, serum creatinine, glucose, triglyceride, total cholesterol, hsCRP (high-sensitivity C-reactive protein) and total energy intake*.

* *p < 0.05*;

** *p < 0.01*.

**Figure 2:**
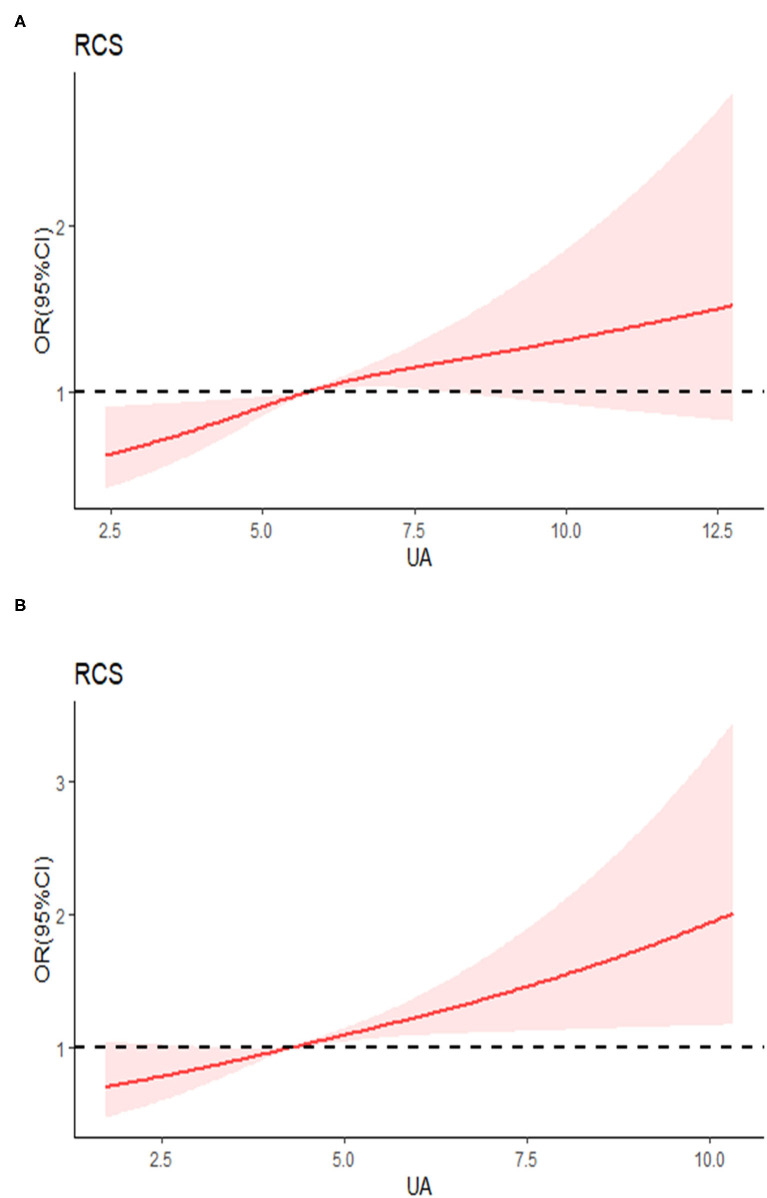
Examination of the dose-response relationship between serum uric acid (μmol/l) and the risk of hypertension by restricted cubic splines model. **(A)** Males, **(B)** females. The restricted cubic splines model adjusted for age, race, living location, body mass index (BMI), alcohol consumption, smoking, diabetes, education, serum creatinine, glucose, triglyceride, total cholesterol, high-sensitivity C-reactive protein (hsCRP), and total energy intake.

The results of the restricted cubic spline dose-response relationship analysis between SUA and hypertension were shown in Figure 2. The prevalence of hypertension increased with elevated SUA concentrations and above relationship was much stronger in females.

In this study, after excluded 2,396 participants who were diagnosed with hypertension and 1,267 participants who were missed followed-up, 4,806 participants were involved in the 2011 follow-up. In 2015, 3,921 participants were involved in the 2015 follow-up. In Table 5, we found that incidence of hypertension among the different uric acid groups increased gradually whether with 2-year or 6-year of follow-up.

**Table 5:** Incidence of hypertension in the different uric acid groups during follow-up in 2011 and 2015.

	**Case/Participants**	**Hypertension (%)**
Uric acid quartiles^†^ In 2011		
Q1	1349/4806	166 (12.3)
Q2	1260/4806	167 (13.3)
Q3	1190/4806	182 (15.3)
Q4	1007/4806	191 (19.0)
*P*		<0.01
In 2015		
Q1	1106/3921	253 (22.9)
Q2	1027/3921	260 (25.3)
Q3	947/3921	249 (26.3)
Q4	841/3921	256 (30.4)
*P*		<0.01

† *Males: Q1 (SUA ≤ 4.86 mg/dL), Q2 (4.86 < SUA ≤ 5.73 mg/dL), Q3 (5.73 < SUA ≤ 6.79 mg/dL), and Q4 (SUA > 6.79 mg/dl)*.

*Females: Q1 (SUA ≤ 3.55 mg/dL), Q2 (3.55 < SUA ≤ 4.29 mg/dL), Q3 (4.29 < SUA ≤ 5.23 mg/dL), and Q4 (SUA > 5.23 mg/dL)*.

Additionally, we further performed stratified analysis by obesity (BMI ≥28 kg/m^2^) or non-obesity and the results are shown in Supplementary Tables S1, S2. This study found that SUA levels were positively associated with the risk of hypertension in the non-obesity group in both males and females, while in obesity participants, above association was not significant.

In stratified analysis by age, the results are shown in Supplementary Tables S3, S4. In males, we found that SUA levels were positively associated with the risk of hypertension for participants between 45 and 60 years old; the ORs (95% CIs) in model 2 were 1.48 (1.05–2.07), 1.33 (0.92–1.92), and 1.57 (1.06–2.32), respectively. In females, above association was significantly for participants over 60 years old; the ORs (95% CIs) in model 2 were 1.28 (0.84–1.94), 1.36 (0.91–2.03), and 1.86 (1.25–2.78), respectively.

## Discussion

In this study, we used data from the CHNS 2009, 2011, and 2015 to explore the correlation between SUA and hypertension in Chinese population; a total of 8,469 individuals (3,973 males and 4,496 females) were included. We found that elevated uric acid levels were positively correlated with the risk of hypertension in both males and females. Whether with 2-year or 6-year follow-up, we found that participants with elevated uric acid levels had a higher incidence of hypertension. In stratified analysis, above association remained significant in non-obesity population, middle-aged men, and elderly women. Restricted cubic spline revealed the dose-response relationship between SUA and hypertension and we also found that above relationship was much stronger in females.

The possible mechanisms of the relationship between uric acid and hypertension have been elucidated in previous studies. Animal model data suggested that elevated uric acid could raise the secretion of renin and then activate the renin-angiotensin system, inhibit intrarenal nitric oxide (NO) synthase expression, and reduce NO release, which led to vasoconstriction and a reversible hypertension. Additionally, previous study found that ischemia-induced xanthine oxidase generates oxidants and uric acid and hyperuricemia was associated with the activation of circulating platelets (24); both uric acid and oxidants were associated with endothelial dysfunction. At this phase, allopurinol or benzbromarone could lower BP and prevent the development of hypertension through reducing uric acid levels. After several weeks, the secretion of monocyte chemoattractant protein 1 and platelet-derived growth factor results in vascular damage, renal afferent arteriolosclerosis, and reduced compliance, which lead to nonreversible hypertension (25, 26). Hyperuricemia was associated with renal afferent arteriolopathy, tubulointerstitial fibrosis, and glomerulosclerosis, which accelerating renal injury and resulting in higher BP (27).

Previous studies have explored the association between SUA and BP and evaluated the effect of urate-lowering therapy on BP. A retrospective cohort study conducted by Kenneth G Saag involved 4,752 young adults, with 20 years of follow-up and they found that participants with higher uric acid had a great hazard of developing hypertension in both males and females compared with the referent group (28). Another study conducted in 2013 involved 44 adolescents who were diagnosed as essential hypertension and with a uric acid >5.5 mg/dl, of whom 20 adolescents received enalapril therapy and 24 adolescents received enalapril plus allopurinol therapy. After 8 weeks follow-up, compared with the enalapril group, both SBP and diastolic blood pressure reduction were greater in the combination therapy group (29). This study also found that higher uric acid levels were associated with an increased risk of hypertension and the effect was more pronounced in women compared with men with the same uric acid levels through the restricted cubic spline curve. During the stratified analysis by obesity, above association became not significant in obesity population, suggesting that the association between uric acid and hypertension was partly mediated through obesity, which was also mentioned in another study (30). During the age stratified analysis, the relationship between SUA and hypertension remained significant for male participants between 45 and 60 years old. Previous studies have also proved that above association was positively significant among people <55 years and ≥40 years (31, 32), which was similar to this study. In females, above association is significant for participants over 60 years old, which might be mediated by the decrease of estrogen levels after menopause (33).

This study has some advantages; first, as a national longitudinal study, we used a large-scale sample among the general Chinese population, which increased the statistical strength and we analyzed this association with different statistical methods and fully adjusted the potential confounding factors. Since the quite difference of SUA between males and females, this study conducted separately in men and women. We used quartiles of SUA by gender to explore the relationship between uric acid and hypertension and restricted cubic splines were used to describe the dose-response relationship between SUA and hypertension. Furthermore, we performed stratified analysis by obesity and age to examine the robustness of above analysis.

The limitations of this study include, primarily, despite the covariates were fully adjusted, we cannot include all the confounding factors due to the limitations of data. Furthermore, the CHNS did not distinguish participants with essential hypertension and secondary hypertension. Although secondary hypertension should be a minority of all the patients with hypertension, the presence of patients with secondary hypertension may influence our analysis. Although triplicate measurements of BP were taken after 10 min at sitting and with at least 1 min between each recording, we could not take into account day-to-day variation of BP because it was measured in a single visit. The CHNS did not provide information on urate-lowering therapy, so the results of uric acid may not represent the true situation and the levels of uric acid might be underestimated. On the other hand, uric acid was measured only once, which was a dynamic but not a static variable. In addition, some antihypertensive drugs can affect the concentrations of uric acid such as hydrochlorothiazide (34) and losartan (35), which can cause bias. However, we are unable to obtain the specific information of antihypertensive drugs for patients with hypertension from the CHNS database.

In conclusion, this study suggests that elevated SUA levels are associated with an increased risk of hypertension in general Chinese adults. Findings of this study indicate the significance of maintaining normal concentration of SUA. We hope that it may provide some information for the prevention and treatment of hypertension.

## Data Availability Statement

The original contributions presented in the study are included in the article/Supplementary Material, further inquiries can be directed to the corresponding authors.

## Ethics Statement

The studies involving human participants were reviewed and the CHNS was approved by the Institutional Review Board at the University of North Carolina at Chapel Hill, the China-Japan Friendship Hospital and the Chinese Center for Disease Control and Prevention's National Institute for Nutrition and Health. The patients/participants provided their written informed consent to participate in this study. Written informed consent was obtained from the individual(s) for the publication of any potentially identifiable images or data included in this article.

## Author Contributions

YH contributed to the conceptualization. KH and HD contributed to the data curation. KH, XH, and HD contributed to the formal analysis. YZ and XZ contributed to the funding acquisition. YH and YY contributed to the investigation. YH, HD, and JW contributed to the methodology. YY contributed to the resources. KH contributed to the software. YZ contributed to the supervision and validation. XH contributed to the visualization. YH contributed to the writing—original draft. YZ and XZ contributed to the writing—review and editing. All the authors helped to perform this study and read and approved the final version of the manuscript.

## Funding

This study was supported by the National Natural Science Foundation of China (Grant Nos. 82071841 and 81901667); CAMS Innovation Fund for Medical Sciences (2020-I2M-2-009); and Clinical and Translational Medical Research fund of Chinese Academy of Medical Sciences (No. 2019XK320013).

## Conflict of Interest

The authors declare that the research was conducted in the absence of any commercial or financial relationships that could be construed as a potential conflict of interest.

## Publisher's Note

All claims expressed in this article are solely those of the authors and do not necessarily represent those of their affiliated organizations, or those of the publisher, the editors and the reviewers. Any product that may be evaluated in this article, or claim that may be made by its manufacturer, is not guaranteed or endorsed by the publisher.
