# Supplementary material for: Serum Uric Acid Might Be Positively Associated With Hypertension in Chinese Adults: An Analysis of the China Health and Nutrition Survey
Source: Front Med (Lausanne). 2022 Jan 5;8:755509. doi: 10.3389/fmed.2021.755509 (PMC8766717; doi:10.3389/fmed.2021.755509)
Supplement: Supplementary file 1 [file Table_1.DOCX]

| **Table S1**. The ORs (95%CIs) of hypertension by serum uric acid in male participants, stratified by BMI, CHNS (N=3973). | | | | | | |
| --- | --- | --- | --- | --- | --- | --- |
|  | Non-Obesity | | | Obesity | | |
|  | Crude ^‡^ | Model 1 ^‡^ | Model 2 ^‡^ | Crude ^‡^ | Model 1 ^‡^ | Model 2 ^‡^ |
| Uric acid quartiles† |  |  |  |  |  |  |
| 1 | 1.00(Ref.) | 1.00(Ref.) | 1.00(Ref.) | 1.00(Ref.) | 1.00(Ref.) | 1.00(Ref.) |
| 2 | 1.08(0.87-1.34) | 1.17(0.93-1.47) | 1.14(0.90-1.43) | 0.80(0.44-1.46) | 0.72(0.38-1.38) | 0.73(0.36-1.49) |
| 3 | 1.24(1.00-1.53) * | 1.39(1.11-1.75) ** | 1.24(0.98-1.57) | 1.39(0.76-2.54) | 1.59(0.83-3.05) | 1.76(0.86-3.61) |
| 4 | 1.52(1.24-1.87) ** | 1.73(1.39-2.16) ** | 1.30(1.01-1.67) * | 1.33(0.73-2.42) | 1.60(0.83-3.07) | 1.94(0.84-4.46) |
| † Quintile ranges:  Non-obesity:1 (SUA≤4.80mg/dL), 2 (4.80＜SUA≤5.66mg/dL), 3 (5.66＜SUA≤6.69mg/dL), 4 (SUA＞6.69mg/dL).  Obesity: 1 (SUA≤5.51mg/dL), 2 (5.51＜SUA≤6.54mg/dL), 3 (6.54＜SUA≤7.50mg/dL), 4 (SUA＞7.50g/dL).  ‡ Calculated using binary logistic regression.  Model 1 adjusted for age and race.  Model 2 adjusted for age, race, living location, BMI, alcohol consumption, smoking, diabetes, education, serum creatinine, glucose, triglyceride, total cholesterol, hsCRP (high-sensitivity C-reactive protein) and total energy intake.  **P* < 0.05; ** *P* < 0.01. | | | | | | |
