# Supplementary material for: Serum Uric Acid Might Be Positively Associated With Hypertension in Chinese Adults: An Analysis of the China Health and Nutrition Survey
Source: Front Med (Lausanne). 2022 Jan 5;8:755509. doi: 10.3389/fmed.2021.755509 (PMC8766717; doi:10.3389/fmed.2021.755509)
Supplement: Supplementary file 2 [file Table_2.DOCX]

| **Table S2**. The ORs (95%CIs) of hypertension by serum uric acid in female participants, stratified by BMI, CHNS (N=4496). | | | | | | |
| --- | --- | --- | --- | --- | --- | --- |
|  | Non-Obesity | | | Obesity | | |
|  | Crude ^‡^ | Model 1 ^‡^ | Model 2 ^‡^ | Crude ^‡^ | Model 1 ^‡^ | Model 2 ^‡^ |
| Uric acid quartiles† |  |  |  |  |  |  |
| 1 | 1.00(Ref.) | 1.00(Ref.) | 1.00(Ref.) | 1.00(Ref.) | 1.00(Ref.) | 1.00(Ref.) |
| 2 | 1.37(1.08-1.74) * | 1.21(0.94-1.56) | 1.15(0.88-1.50) | 1.58(0.94-2.64) | 1.42(0.82-2.45) | 1.35(0.76-2.40) |
| 3 | 1.75(1.39-2.21) ** | 1.35(1.05-1.73) * | 1.18(0.91-1.53) | 1.49(0.90-2.49) | 1.21(0.70-2.08) | 1.08(0.60-1.95) |
| 4 | 3.46(2.78-4.30) ** | 2.01(1.58-2.56) ** | 1.52(1.16-1.99) ** | 2.22(1.32-3.72) ** | 1.69(0.97-2.93) | 1.43(0.74-2.76) |
| † Quintile ranges:  Non-obesity: 1 (SUA≤3.51mg/dL), 2 (3.51＜SUA≤4.22mg/dL), 3 (4.22＜SUA≤5.11mg/dL), 4 (SUA＞5.11mg/dL).  Obesity: 1 (SUA≤4.14mg/dL), 2 (4.14＜SUA≤5.06mg/dL), 3 (5.06＜SUA≤5.99mg/dL), 4 (SUA＞5.99mg/dL).  ‡ Calculated using binary logistic regression.  Model 1 adjusted for age and race.  Model 2 adjusted for age, race, living location, BMI, alcohol consumption, smoking, diabetes, education, serum creatinine, glucose, triglyceride, total cholesterol, hsCRP (high-sensitivity C-reactive protein) and total energy intake.  **P* < 0.05; ***P* < 0.01. | | | | | | |
