# Supplementary material for: Serum Uric Acid Might Be Positively Associated With Hypertension in Chinese Adults: An Analysis of the China Health and Nutrition Survey
Source: Front Med (Lausanne). 2022 Jan 5;8:755509. doi: 10.3389/fmed.2021.755509 (PMC8766717; doi:10.3389/fmed.2021.755509)
Supplement: Supplementary file 3 [file Table_3.DOCX]

| **Table S3**. The ORs (95%CIs) of hypertension by serum uric acid in male participants, stratified by age, CHNS (N=3973). | | | | | | | | | | |
| --- | --- | --- | --- | --- | --- | --- | --- | --- | --- | --- |
|  |  | 18≤Age＜45 Years | |  | 45≤Age＜60 Years | |  | | Age≥60 Years | |
|  | Crude^‡^ | Model 1^‡^ | Model 2^‡^ | Crude^‡^ | Model 1^‡^ | Model 2^‡^ | Crude^‡^ | Model 1^‡^ | | Model 2^‡^ |
| Uric acid quartiles† |  |  |  |  |  |  |  |  | |  |
| 1 | 1.00(Ref.) | 1.00(Ref.) | 1.00(Ref.) | 1.00(Ref.) | 1.00(Ref.) | 1.00(Ref.) | 1.00(Ref.) | 1.00(Ref.) | | 1.0 (Ref.) |
| 2 | 1.25(0.78-2.00) | 1.33(0.83-2.13) | 1.28(0.78-2.13) | 1.57(1.14-2.16) ** | 1.60(1.17-2.21) ** | 1.48(1.05-2.07) * | 0.95(0.68-1.33) | 0.91(0.65-1.29) | | 0.81(0.57-1.17) |
| 3 | 1.04(0.65-1.68) | 1.20(0.74-1.95) | 1.04(0.62-1.75) | 1.64(1.18-2.29) ** | 1.70(1.22-2.38) ** | 1.33(0.92-1.92) | 1.48(1.07-2.05) * | 1.48(1.06-2.06) * | | 1.21(0.84-1.73) |
| 4 | 1.79(1.15-2.78) * | 1.90(1.21-2.97) ** | 1.37(0.80-2.35) | 2.19(1.59-3.00) ** | 2.23(1.62-3.07) ** | 1.57(1.06-2.32) * | 1.96(1.40-2.74) ** | 1.90(1.36-2.68) ** | | 1.26(0.84-1.89) |
| † Quintile ranges:1 (SUA≤4.86mg/dL), 2 (4.86＜SUA≤5.73mg/dL), 3 (5.73＜SUA≤6.79mg/dL), 4 (SUA＞6.79mg/dL).  ‡ Calculated using binary logistic regression.  Model 1 adjusted for age and race.  Model 2 adjusted for age, race, living location, BMI, alcohol consumption, smoking, diabetes, education, serum creatinine, glucose, triglyceride, total cholesterol, hsCRP (high-sensitivity C-reactive protein) and total energy intake.  **P*< 0.05; ***P*< 0.01. | | | | | | | | | | |
