# Supplementary material for: Serum Uric Acid Might Be Positively Associated With Hypertension in Chinese Adults: An Analysis of the China Health and Nutrition Survey
Source: Front Med (Lausanne). 2022 Jan 5;8:755509. doi: 10.3389/fmed.2021.755509 (PMC8766717; doi:10.3389/fmed.2021.755509)
Supplement: Supplementary file 4 [file Table_4.DOCX]

| **Table S4**. The ORs (95%CIs) of hypertension by serum uric acid in female participants, stratified by age, CHNS (N=4496). | | | | | | | | | | |
| --- | --- | --- | --- | --- | --- | --- | --- | --- | --- | --- |
|  |  | 18≤Age＜45 Years | |  | 45≤Age＜60 Years | |  | | Age≥60 Years | |
|  | Crude^‡^ | Model 1^‡^ | Model 2^‡^ | Crude^‡^ | Model 1^‡^ | Model 2^‡^ | Crude^‡^ | Model 1^‡^ | | Model 2^‡^ |
| Uric acid quartiles† |  |  |  |  |  |  |  |  | |  |
| 1 | 1.00(Ref.) | 1.00(Ref.) | 1.00(Ref.) | 1.00(Ref.) | 1.00(Ref.) | 1.00(Ref.) | 1.00(Ref.) | 1.00(Ref.) | | 1.0 (Ref.) |
| 2 | 1.10(0.67-1.82) | 1.16(0.69-1.92) | 1.13(0.66-1.97) | 1.12(0.81-1.56) | 1.08(0.77-1.51) | 0.93(0.65-1.31) | 1.28(0.87-1.88) | 1.33(0.90-1.96) | | 1.28(0.84-1.94) |
| 3 | 1.20(0.72-2.00) | 1.33(0.79-2.24) | 1.24(0.70-2.18) | 1.37(0.99-1.90) | 1.26(0.91-1.76) | 0.94(0.66-1.34) | 1.48(1.03-2.14) * | 1.56(1.08-2.27) * | | 1.36(0.91-2.03) |
| 4 | 1.86(1.09-3.17) * | 2.04(1.18-3.53) * | 1.14(0.57-2.28) | 2.24(1.64-3.07) ** | 2.07(1.50-2.85) ** | 1.28(0.88-1.87) | 2.54(1.80-3.58) ** | 2.54(1.79-3.60) ** | | 1.86(1.25-2.78) ** |
| † Quintile ranges:1 (SUA≤3.55mg/dL), 2 (3.55＜SUA≤4.29mg/dL), 3 (4.29＜SUA≤5.23mg/dL), 4 (SUA＞5.23mg/dL).  ‡ Calculated using binary logistic regression.  Model 1 adjusted for age and race.  Model 2 adjusted for age, race, living location, BMI, alcohol consumption, smoking, diabetes, education, serum creatinine, glucose, triglyceride, total cholesterol, hsCRP (high-sensitivity C-reactive protein) and total energy intake.  **P* < 0.05; ***P* < 0.01. | | | | | | | | | | |
